# Lobe-Specific Analysis of Sublobar Lung Resection for NSCLC Patients with Tumors ≤ 2 cm

**DOI:** 10.3390/cancers14133265

**Published:** 2022-07-04

**Authors:** Xi Lei, Ning Zhou, Hao Zhang, Tong Li, Fan Ren, Bo Zhang, Xiongfei Li, Lingling Zu, Zuoqing Song, Song Xu

**Affiliations:** 1 Department of Lung Cancer Surgery, Tianjin Medical University General Hospital, Tianjin 300052, China; woshileixi@outlook.com (X.L.); xiaozhouning@outlook.com (N.Z.); zcyzhanghao@163.com (H.Z.); tjzyylitong@126.com (T.L.); rfdream@163.com (F.R.); bozhang725@163.com (B.Z.); feacaution@outlook.com (X.L.); zulingling@tmu.edu.cn (L.Z.); thoracic_expert@aliyun.com (Z.S.); 2 Tianjin Key Laboratory of Lung Cancer Metastasis and Tumor Microenvironment, Lung Cancer Institute, Tianjin Medical University General Hospital, Tianjin 300052, China

**Keywords:** lobe-specific, sublobar resection, segmentectomy, wedge resection, early-stage NSCLC

## Abstract

**Simple Summary:**

With the increase of the use of sublobar resection, the optimal strategy for early-stage NSCLC patients is essential. Previous studies showed tumors in different lobes are associated with different prognoses after radiotherapy and immunotherapy, inspiring us that each lobes had a different prognosis. Thus, we investigated the best surgical procedure for sublobar resection in patients with NSCLC ≤ 2 cm based on a lobe-specific analysis using propensity score matching. The results of this study will make it easier for surgeons to screen out patients with NSCLC ≤ 2 cm for segmentectomy or wedge resection, although this must be validated in larger cohorts.

**Abstract:**

(1) Background: Sublobar resection can be used as an alternative surgical strategy for early-stage non-small-cell lung cancer (NSCLC) patients. However, the choice between wedge resection and segmentectomy remains contentious. In this study, we investigated the optimal surgical procedure for sublobar resection in patients with NSCLC ≤ 2 cm with a lobe-specific analysis; (2) Methods: Data for patients with T1N0M0 with a diameter of ≤2 cm who had undergone sublobar resection were retrieved. Propensity score matching (PSM) was used to reduce the inherent bias, and the Kaplan–Meier method and log-rank tests were used to assess the differences in survival; (3) Results: A total of 1882 patients were identified after the PSM. Patients with NSCLC ≤ 2 cm who had undergone segmentectomy showed better survival than those who had undergone wedge resection. However, when NSCLC was ≤1 cm, there was no significant difference in OS between the two groups. This demonstrated an OS advantage of segmentectomy over wedge resection for patients with NSCLC tumors of 1–2 cm (*p* = 0.024). Further analysis indicated that this survival benefit was only observed in patients with right upper NSCLC of 1–2 cm, but not with NSCLC in the other four lobes; (4) Conclusions: Segmentectomy showed a greater survival benefit than wedge resection only in patients with NSCLC of 1–2 cm, particularly those with primary tumors in the right upper lobe. Therefore, we propose a lobe-specific sublobar resection strategy for early-stage NSCLC patients (tumors of 1–2 cm) who cannot tolerate lobectomy.

## 1. Introduction

Surgery is widely accepted as the first choice for the cure of patients with early-stage non-small-cell lung cancer (NSCLC) [1]. Lobectomy has been the standard surgical treatment for patients with stage I NSCLC because a landmark randomized controlled trial by the Lung Cancer Study Group provided high-quality evidence for lobectomy at the end of the last century [2]. Compared with lobectomy, the main advantages of sublobar resection are the reduction of perioperative morbidity and the preservation of postoperative pulmonary function [3,4]. Recent retrospective studies have demonstrated that survival is similar after both sublobar resection and lobectomy [5,6,7,8]. Meanwhile, with the increased detection of small tumors and the association between the appearance of ground-glass opacity and a good histological type, the use of sublobar resection has increased [9].

Segmentectomy and wedge resection are two types of sublobar resection; the biggest difference between these two methods is that segmentectomy requires the oncology standard of lobectomy, such as the anatomy of the separation of pulmonary segment veins, arteries, and bronchi, and the better removal of lung parenchymal tissue. However, the choice between wedge resection and segmentectomy as the superior sublobar resection method in NSCLC patients with tumors ≤ 2 cm remains contentious due to the contradictory results [10,11,12,13]. Therefore, selecting the optimal extent of resection in terms of the potential long-term outcome and the risk of perioperative complications and death is difficult for surgeons, indicating that the optimal sublobar resection strategy for early-stage NSCLC patients is essential.

Previous studies have indicated that lower lobectomy is associated with significantly poorer overall survival (OS) than upper lobectomy [14,15,16,17,18]. However, none of those studies considered the effects of the lobular site of the tumor on the prognosis when recommending the optimal surgical procedure for sublobar resection. Therefore, we investigated the best surgical procedure for sublobar resection in patients with NSCLC ≤ 2 cm based on a lobe-specific analysis using propensity score matching.

## 2. Materials and Methods

### 2.1. Patient Selection

Patients with NSCLC diagnosed between 1975 and 2016 were extracted from the “Incidence-SEER 18 Regs Custom Data (with additional treatment fields), November 2018 Sub [1975–2016 varying]” (SEER stat 8.2.9.2), according to the “Site Recode ICD-0-3/WHO 2008” and “ICD-0-3 His/Behav, malignant”. Patients were included in our study if they met the following inclusion criteria: (1) pathologically confirmed primary T1N0M0 NSCLC of stage IA (≤2 cm in size); (2) a history of segmentectomy or wedge resection. We excluded any patients who reported undergoing perioperative radiation therapy or chemotherapy or in whom the location of the primary tumor was unclear. Similarly, patients for whom the pathology results were only confirmed at autopsy or death and those with other primary cancers in their lifetimes were also excluded.

The demographic data of the patients, the characteristics of tumors, survival, vital status, and the surgery they underwent were collected from the SEER database. In this study, the histological subtypes were classified as lung squamous cell carcinoma (LUSC), lung adenocarcinoma (LUAD), and other carcinoma (OC).

### 2.2. Statistical Analysis

We divided all the patients into two groups according to the surgical procedure. We used propensity score matching (PSM) to reduce the inherent bias of retrospective studies. The distributions of variables were analyzed with an χ^2^ test, or with Fisher’s exact test for categorical variables and a t test for continuous variables, using SPSS version 26.0 (IBM Corporation, Armonk, NY, USA). Univariate and multivariate analyses were performed with a Cox regression analysis using SPSS (IBM Corporation, Armonk, NY, USA), version 26.0, and variables were excluded sequentially if the α error was >0.05. The Kaplan–Meier method was used to determine OS after segmentectomy or wedge resection in patients with NSCLC ≤ 2 cm. A log-rank test was used to compare the survival curves. All tests were two-sided, and a *p* value of <0.05 was considered significant.

## 3. Results

### 3.1. Patient Characteristics

**Figure 1:**
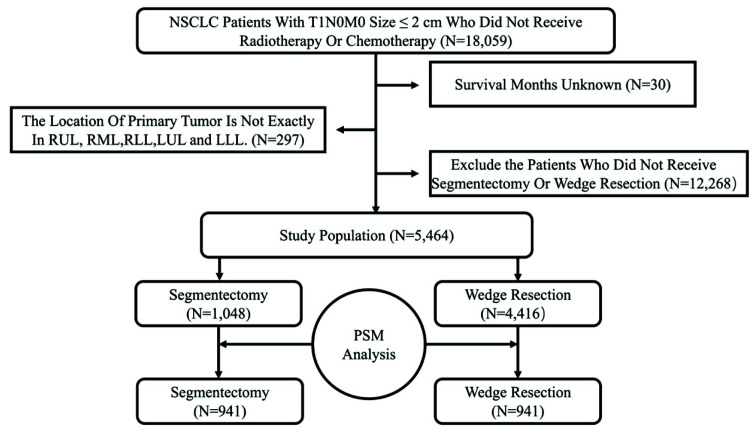
Selection of eligible patients.

Before PSM, a total of 5464 patients with T1N0M0 NSCLC (tumor size ≤ 2 cm) were enrolled in the study. A complete flow chart of the selection process is shown in Figure 1. All the characteristics are summarized in Table 1.

As shown in Table 1, there were no differences in age, sex, race, or histological type between the segmentectomy and wedge resection groups (*p* = 0.871, 0.240, 0.869, and 0.163, respectively), whereas statistically significant differences existed in tumor location, tumor size, and the number of resected LNs (*p* < 0.001). Among the originally enrolled patients, 1048 (19.18%) and 4416 (80.92%) underwent segmentectomy and wedge resection, respectively, and 57.21% of the enrolled patients had undergone LN dissection. Wedge resection seemed to be more commonly performed in patients with a tumor size ≤ 2 cm, but as the tumor grew larger, segmentectomy was more frequently performed. For patients with a tumor size ≤ 2 cm, LUAD (the majority histological type) accounted for 57% of tumors, whereas LUSC and OC accounted for 19.13% and 23.81%, respectively. Interestingly, in this study, more tumors were located in the upper lobes than in the lower or middle lobes: right upper lobe (RUL) 1683, right middle lobe (RML) 303, right lower lobe (RLL) 974, left upper lobe (LUL) 1544, and left lower lobe (LLL) 960.

To reduce the inherent bias, we considered age, sex, race, histological type, tumor location, tumor size, and the number of resected LNs as matched confounding factors to perform PSM. After PSM, a total of 1882 patients were enrolled in the study, including 941 patients in the segmentectomy group and 941 patients in the wedge resection group. As summarized in Table 1, after PSM, no significant differences were detected in the characteristics of these two groups.

### 3.2. Surgical Outcome Analysis

**Figure 2:**
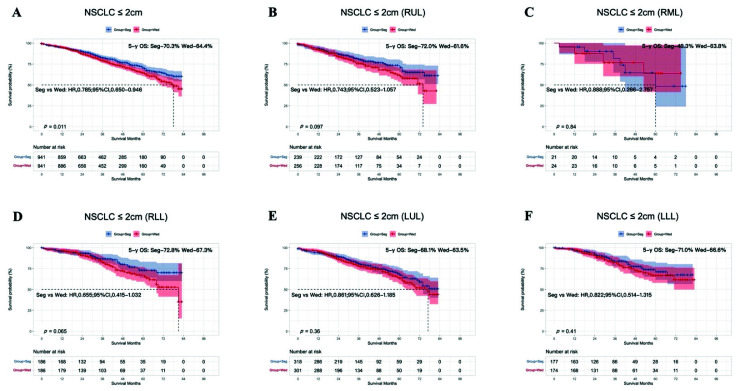
Kaplan–Meier survival curves after segmentectomy or wedge resection in NSCLC patients with tumors ≤ 2 cm after PSM (**A**). Survival curves for the RUL (**B**), RML (**C**), RLL (**D**), LUL (**E**), and LLL (**F**) after PSM.

Among all the patients before PSM, a log-rank test revealed that patients who underwent segmentectomy had a significantly better OS than those who underwent wedge resection (HR, 0.774; 95% CI, 0.670–0.893; 5-year OS: segmentectomy 69.9%, wedge resection 62.9%; *p* < 0.001; Figure S1A). After PSM, OS was still significantly better in the segmentectomy group than in the wedge resection group (HR, 0.785; 95% C1, 0.650–0.946; 5-year OS: segmentectomy 70.3%, wedge resection 64.4%; *p* = 0.011; Figure 2A). However, after we divided patients according to the location of the primary tumor, segmentectomy was not associated with significantly better OS than wedge resection (RUL, *p* = 0.097; RML, *p* = 0.84; RLL, *p* = 0.065; LUL, *p* = 0.36; LLL, *p* = 0.41; Figure 2B–F).

### 3.3. Subgroup Analysis According to Tumor Size

**Figure 3:**
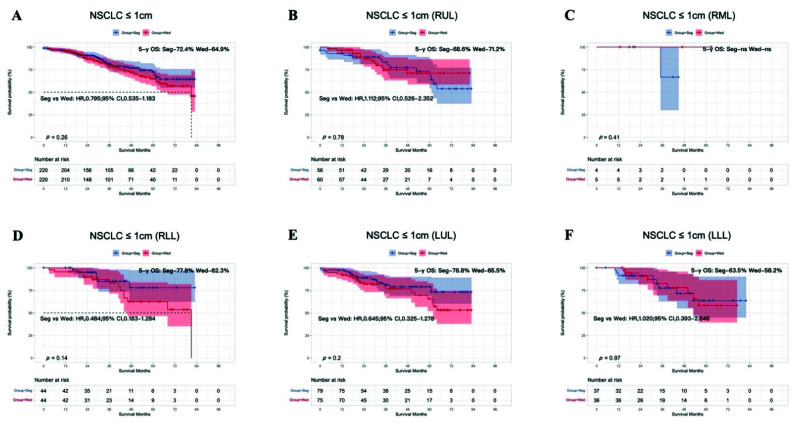
Kaplan–Meier survival curves after segmentectomy or wedge resection in NSCLC patients with tumors ≤ 1 cm after PSM (**A**). Survival curves for the RUL (**B**), RML (**C**), RLL (**D**), LUL (**E**), and LLL (**F**) after PSM.

We investigated the survival analysis according to the tumor size subclassification (T < 1 cm or T = 1–2 cm in size). In the T < 1 cm group, before PSM was performed, segmentectomy showed a significant advantage for OS compared with wedge resection (HR, 0.731; 95% CI, 0.543–0.984; 5-year OS: segmentectomy 73.4%, wedge resection 63.7%; *p* = 0.038; Figure S1B). However, after PSM, OS after segmentectomy was similar to that after wedge resection (HR, 0.795; 95% CI, 0.535–1.183; 5-year OS: segmentectomy 72.4%, wedge resection 64.9%; *p* = 0.26; Figure 3A). When T < 1 cm group was divided into five lobar subgroups, OS after segmentectomy and wedge resection was similar in the five lobar subgroups (RUL, *p* = 0.78; RML, *p* = 0.41; RLL, *p* = 0.14; LUL, *p* = 0.2; LLL, *p* = 0.97; Figure 3B–F).

**Figure 4:**
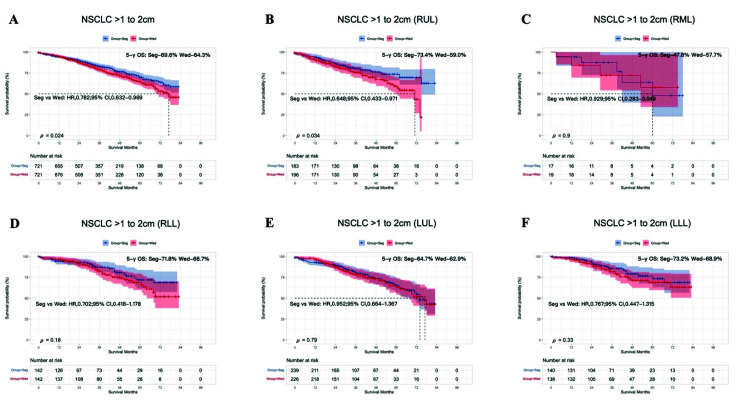
Kaplan–Meier survival curves after segmentectomy or wedge resection in NSCLC patients with tumors of 1–2 cm after PSM (**A**). Survival curves for the RUL (**B**), RML (**C**), RLL (**D**), LUL (**E**), and LLL (**F**) after PSM.

The results for the T = 1–2 cm group were similar before (HR, 0.780; 95% CI, 0.661–0.920; 5-year OS: segmentectomy 68.8%, wedge resection 62.5%, *p* < 0.001, Figure S1C) and after (HR, 0.782: 95% Cl, 0.632–0.969; 5-year OS: segmentectomy 69.6%, wedge resection 64.3%, *p* = 0.024; Figure 4A) PSM, in that segmentectomy produced better OS than wedge resection.

Analysis of the lobar subgroups showed that for tumors occurring in most lobes, there was no difference in OS after segmentectomy or wedge resection (RML, *p* = 0.9; RLL, *p* = 0.18; LUL, *p* = 0.79; LLL, *p* = 0.33; Figure 4C–F). However, patients with tumors in RUL showed better OS after segmentectomy than after wedge resection (HR, 0.648; 95% Cl, 0.433–0.971; 5-year OS: segmentectomy 73.4%, wedge resection 59.0%, *p* = 0.034; Figure 4B).

### 3.4. Subgroup Analysis According to Age after PSM

Based on the median age (70 years), we divided the patients into younger and older groups: ≤70 years and >70 years, respectively. As shown in Figure S2, there was no significant difference in OS after segmentectomy or wedge resection in the younger group (*p* = 0.27; Figure S2A). However, in the older group, a significantly greater survival benefit after segmentectomy than after wedge resection was detected with a log-rank test (HR, 0.761; 95% Cl, 0.595–0.974; 5-year OS: segmentectomy 63.1%, wedge resection 56.2%; *p* = 0.029; Figure S2B).

### 3.5. Subgroup Analysis According to Histological Type after PSM

LUSC and LUAD accounted for 80% of the tumors in our analysis, so patients were divided according to LUSC, LUAD, and OC for a further survival analysis. In patients with LUSC or LUAD, there was no statistical difference in OS after either type of resection (*p* = 0.68, *p* = 0.15, respectively; Figure S3A,B). Interestingly, segmentectomy showed a significant survival benefit in the OC group (HR, 0.389; 95% CI, 0.232–0.655; 5-year OS: segmentectomy 86.1%, wedge resection 57.5%, *p* < 0.001; Figure S3C).

### 3.6. Subgroup Analysis According to Sex after PSM

Survival was also investigated according to sex, as shown in Figure S4A,B. Males showed better OS after segmentectomy than after wedge resection (HR, 0.692; 95% C1, 0.526–0.911; 5-year OS: segmentectomy 63.3%, wedge resection 51.5%, *p* = 0.008), whereas this benefit was not observed in the female group (*p* = 0.38).

### 3.7. Cox Regression Analysis

**Figure 5:**
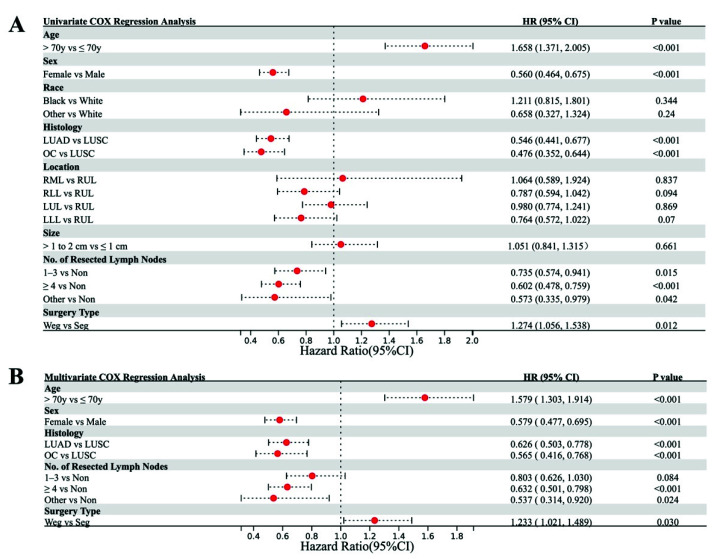
Univariate (**A**) and multivariate (**B**) Cox regression analyses of factors affecting overall survival.

A Cox regression analysis was performed to identify any potentially confounding factors related to OS. The hazard ratios, *p* values, and details of the 95% confidence intervals are summarized in Table S1. The univariate analysis indicated that older age, male sex, LUSC, no LN dissection, and wedge resection were associated with worse OS, whereas race and tumor location and tumor size were not associated with worse OS (Figure 5A). Similarly, the multivariate Cox regression analysis showed that greater age, male sex, LUSC, no LN dissection, and wedge resection were associated with poor OS (Figure 5B).

## 4. Discussion

In this study, 5464 patients, including 1048 (19.18%) who underwent segmentectomy and 4416 (80.92%) who underwent wedge resection were enrolled. Before PSM, OS was significantly better after segmentectomy than after wedge resection among all the patients. However, a subgroup analysis according to tumor size after PSM showed that this survival benefit was only conferred on patients with NSCLC tumors of 1–2 cm, and not in those with tumors < 1 cm. Interestingly, Dai et al. published a study that indicated that in patients for whom lobectomy was unsuitable, segmentectomy should be recommended for NSCLC with tumors of 1–2 cm, whereas the choice between segmentectomy and wedge resection for NSCLC ≤ 1 cm should be based on the surgical skills available and the patient profile [19]. A meta-analysis also supported part of our results, in that wedge resection was not inferior to segmentectomy in patients with NSCLC ≤ 1 cm [20]. However, that analysis also suggested that segmentectomy can achieve better OS than wedge resection for stage IA NSCLC tumors ≤ 2 cm, which differs from our result after PSM. This discrepancy may be attributable to the fact that in our study, any bias caused by age, sex, race, histological type, tumor location, tumor size, or the number of resected LNs was reduced with PSM. As shown in Figure 2A, 5-year OS for T1N0M0 patients with tumor ≤ 2 cm was around 70% after receiving segmentectomy or wedge resection, which was much shorter than previous reports. According to previous study [21], for patients with pure ground nodule or part-solid nodules, the 5-year OS was better than that of patients with pure solid nodules (nearly 100% vs. 80%). Furthermore, the study by Aritoshi et al. [22] showed that 5-year OS for patients with tumor ≤ 1 cm, ≤ 2 cm, and ≤ 3 cm was 87.9%, 85.9%, and 73.7, respectively. It was obvious that the larger the solid tumor was, the shorter 5-year OS was. Thus, small nodules including pure solid tumors, part-solid tumor, and pure ground glass nodules were all enrolled in this study due to the discrepancy of related information in SEER database, which would lead to the difference between our study and previous studies. Our univariate analysis indicated that older age, male sex, LUSC, no LN dissection, and wedge resection were associated with worse OS, and a multivariate analysis also showed that older age, male sex, LUSC, no LN dissection, and wedge resection were associated with poor OS. These results are completely consistent with a previous study by Bo et al., who examined the prognostic significance of the histological type of NSCLC tumors ≤ 2 cm [23].

To the best of our knowledge, this study is the first to discuss the difference between segmentectomy and wedge resection for NSCLC in different lung lobes. Although several studies have shown that NSCLC patients with primary tumors located in different lobes have different prognoses after the same treatment, including surgery and radiotherapy [14,15,16,17,18], none of those studies analyzed the prognosis according to the different individual lobes. Another study, in which 19,702 patients with stage I NSCLC were enrolled from the California Cancer Registry (CCR) database, demonstrated survival differences among patients with tumors in five lobar locations after surgical resection [15]. However, a subgroup analysis revealed that the “upper” group (RUL and LUL) showed better OS than the “non-upper” group (RML, RLL, and LLL) (*p* < 0.005). Similarly, a study by Kazuhiro et al., in which 422 cases treated at Yamaguchi University Hospital between January 2007 and October 2015 were reviewed retrospectively, demonstrated that lower lobectomy was associated with significantly worse recurrence-free survival and OS than upper lobectomy (including middle lobectomy; *p* < 0.05) [14]. In the present study, a lobe-specific analysis was conducted to determine the precise sublobar surgical procedure for patients with NSCLC ≤ 2 cm. In the lobe-specific analysis of the T < 1 cm group, the survival benefit of wedge resection was not inferior to that of segmentectomy, consistent with the lobe-nonspecific analysis. In contrast, in the subgroup analysis of the T = 1–2 cm group, segmentectomy only produced better OS than wedge resection when the primary tumor was located in RUL, indicating that surgical management may be based not only on the tumor size but also on its location. However, the mechanism underlying the correlation between the tumor location and the prognosis is still unknown, and requires further research.

There were several limitations to our study. Firstly, as a retrospective study, there was still inevitable bias, even though PSM was performed. Secondly, the number of NSCLC patients undergoing sublobar resection was not very large, especially those with NSCLC located in RML. It was demonstrated that only 45 patients with tumor in RML were enrolled in our study, of which there were 9 patients with tumor ≤ 1 cm and 36 patients with tumor >1 to 2 cm. Thus, results in RML needed to be validated in a larger sample size cohort due to deficiency in sample size. Thirdly, although the time span narrowed down compared to original dataset (1975–2016) after we chose a tag named CS tumor size (2004–2015) to screen out the patients whose primary tumor was ≤2 cm, the progression of minimally surgical techniques may influence the preference of surgical approach as well. However, the study by Yang et al. revealed that minimally invasive approaches to lobectomy result in similar long-term survival as thoracotomy [24]. Last but not least, the SEER database provided no information on the radiological features of the tumors (such as the solid ratios for tumors or the molecular phenotypes), pulmonary function, and specific lung segment (single segment or combined segments). These factors may also influence the choice of surgical procedure. For example, segmentectomy and wedge resection was an alternative choice when lobectomy was not appropriate due to poor pulmonary function. Although, compared to lobectomy, sublobar resection could preserve the pulmonary function as much as possible, pulmonary function loss after segmentectomy was still greater than after wedge resection [25], which would influence the final result.

## 5. Conclusions

In conclusion, in NSCLC tumors ≤2 cm, segmentectomy showed a survival benefit relative to wedge resection only for NSCLC tumors of 1–2 cm, and particularly for primary tumors in RUL. Therefore, for patients with early-stage NSCLC (1–2 cm) who cannot tolerate lobectomy, we propose a lobe-specific sublobar resection strategy, although this recommendation must be validated in larger cohorts.

## Figures and Tables

**Table 1:** Baseline characteristics of NSCLC patients with size ≤ 2 cm.

Characteristics	Before PSM				After PSM			
	Estimate	Segmentectomy	Wedge Resection	*p* Value	Estimate	Segmentectomy	Wedge Resection	*p* Value
	5464 (100%)	1048 (19.18%)	4416 (80.92%)		1882 (100.00%)	941 (50.00%)	941 (50.00%)	
Age				0.871				0.356
≤70	2923 (53.50%)	563 (10.30%)	2360 (43.19%)		986 (52.39%)	503 (26.73%)	483 (25.66%)	
>70	2541 (46.5%)	485 (8.88%)	2056 (37.63%)		896 (47.61%)	438 (23.27%)	458 (24.34%)	
Sex				0.240				0.337
Male	2115 (38.71%)	389 (7.12%)	1726 (31.59%)		676 (35.92%)	328 (17.43%)	348 (18.49%)	
Female	3349 (61.29%)	659 (12.06%)	2690 (49.23%)		1206 (64.08%)	613 (32.57%)	593 (31.51%)	
Race				0.869				1.000
White	4780 (87.48%)	920 (16.84%)	3860 (70.64%)		1734 (92.14%)	867 (46.07%)	867 (46.07%)	
Black	411 (7.52%)	79 (1.45%)	332 (6.08%)		88 (4.68%)	44 (2.34%)	44 (2.34%)	
Other	273 (5.00%)	49 (0.90%)	224 (4.10%)		60 (3.19%)	30 (1.59%)	30 (1.59%)	
Histologic Type				0.163				0.320
LUSC	1045 (19.13%)	183 (3.35%)	862 (15.78%)		332 (17.64%)	156 (8.29%)	176 (9.35%)	
LUAD	3118 (57.06%)	624 (11.42%)	2494 (45.64%)		1178 (62.59%)	589 (31.30%)	589 (31.30%)	
OC	1301 (23.81%)	241 (4.41%)	1060 (19.40%)		372 (19.77%)	196 (10.41%)	176 (9.35%)	
Location				<0.001				0.865
Right Upper Lobe	1683 (30.80%)	251 (4.59%)	1432 (26.21%)		495 (26.30%)	239 (12.70%)	256 (13.60%)	
Right Middle Lobe	303 (51.55%)	26 (0.48%)	277 (5.07%)		45 (2.39%)	21 (1.12%)	24 (1.28%)	
Right Lower Lobe	974 (17.83%)	209 (3.83%)	765 (14.00%)		372 (19.77%)	186 (9.88%)	186 (9.88%)	
Left Upper Lobe	1544 (28.26%)	351 (6.42%)	1193 (21.83%)		619 (32.89%)	318 (16.90%)	301 (15.99%)	
Left Lower Lobe	960 (17.57%)	211 (3.86%)	749 (13.71%)		351 (18.65%)	177 (9.40%)	174 (9.25%)	
Tumor Size				<0.001				1.000
≤1 cm	1730 (31.66%)	256 (4.69%)	1474 (26.98%)		440 (23.38%)	220 (11.69%)	220 (11.69%)	
>1 cm to 2 cm	3734 (68.34%)	792 (14.49%)	2942 (53.84%)		1442 (76.62%)	721 (38.31%)	721 (38.31%)	
No. of Resected Lymph Nodes				<0.001				0.845
0	2338 (42.79%)	208 (3.81%)	2130 (38.98%)		385 (20.46%)	196 (10.41%)	189 (10.04%)	
>1 to 3	1343 (24.58%)	269 (4.92%)	1074 (19.66%)		501 (26.62%)	250 (13.28%)	251 (13.34%)	
≥4	1574 (28.81%)	523 (9.57%)	1051 (19.23%)		925 (49.15%)	463 (24.60%)	462 (24.55%)	
Other	209 (3.83%)	48 (0.88%)	161 (2.95%)		71 (3.77%)	32 (1.70%)	39 (2.07%)	

## Data Availability

SEER stat can be downloaded from the Surveillance, Epidemiology, and End Results Program (https://seer.cancer.gov/data-software/ (accessed on 15 January 2022).

## Supplementary Materials

The following supporting information can be downloaded at: https://www.mdpi.com/article/10.3390/cancers14133265/s1, Figure S1: Kaplan–Meier survival curves after segmentectomy or wedge resection in NSCLC patients with tumors ≤ 2 cm, ≤ 1 cm, and 1–2 cm before PSM; Figure S2: Subgroup analysis of overall survival after PSM following segmentectomy or wedge resection in NSCLC patients with tumors ≤ 2 cm, according to age: ≤70 years and >70 years; Figure S3: Subgroup analysis of overall survival after PSM following segmentectomy or wedge resection in NSCLC patients with tumors ≤ 2 cm according to histological type: LUSC, LUAD, and OC. LUSC, Lung squamous cell carcinoma; LUAD, Lung adenocarcinoma; OC, Other carcinoma; Figure S4: Subgroup analysis of overall survival after PSM following segmentectomy or wedge resection in NSCLC patients with tumors ≤ 2 cm, according to sex: male and female; Table S1: Details of Cox Regression Analyses.
