# Supplementary material for: Lobe-Specific Analysis of Sublobar Lung Resection for NSCLC Patients with Tumors ≤ 2 cm
Source: Cancers (Basel). 2022 Jul 4;14(13):3265. doi: 10.3390/cancers14133265 (PMC9265391; doi:10.3390/cancers14133265)
Supplement: Supplementary file 1 [file cancers-14-03265-s001.zip › supplementary materials/table S1.pdf]

Table S1. Details of COX Regression Analysis

Univariate COX Regression Analysis		HR (95% CI)	P value
**Age**			
	> 70y vs ≤ 70y	1.658 (1.371, 2.005)	<0.001
**Sex**			
	Female vs Male	0.560 (0.464, 0.675)	<0.001
**Race**			0.309
	Black vs White	1.211 (0.815, 1.801)	0.344
	Other vs White	0.658 (0.327, 1.324)	0.240
**Histology**			<0.001
	LUAD vs LUSC	0.546 (0.441, 0.677)	<0.001
	OC vs LUSC	0.476 (0.352, 0.644)	<0.001
**Location**			0.192
	RML vs RUL	1.064 (0.589, 1.924)	0.837
	RLL vs RUL	0.787 (0.594, 1.042)	0.094
	LUL vs RUL	0.980 (0.774, 1.241)	0.869
	LLL vs RUL	0.764 (0.572, 1.022)	0.070
**Size**			
	> 1 to 2 cm vs ≤ 1 cm	1.051 (0.841, 1.315)	0.661
**No. of Resected Lymph Nodes**			<0.001
	1–3 vs Non	0.735 (0.574, 0.941)	0.015
	≥ 4 vs Non	0.602 (0.478, 0.759)	<0.001
	Other vs Non	0.573 (0.335, 0.979)	0.042
**Surgery Type**			
	Wed vs Seg	1.274 (1.056, 1.538)	0.012

Multivariate COX Regression Analysis		HR (95% CI)	P value
**Age**			
	> 70y vs ≤ 70y	1.579 (1.303, 1.914)	<0.001
**Sex**			
	Female vs Male	0.579 (0.477, 0.695)	<0.001
**Histology**			<0.001
	LUAD vs LUSC	0.626 (0.503, 0.778)	<0.001
	OC vs LUSC	0.565 (0.416, 0.768)	<0.001
**No. of Resected Lymph Nodes**			0.001
	1–3 vs Non	0.803 (0.626, 1.030)	0.084
	≥ 4 vs Non	0.632 (0.501, 0.798)	<0.001
	Other vs Non	0.537 (0.314, 0.920)	0.024
**Surgery Type**			
	Wed vs Seg	1.233 (1.021, 1.489)	0.030
